# Iron chelation in treatment of superficial siderosis

**Published:** 2018-10-07

**Authors:** Eda Derle

**Affiliations:** Department of Neurology, School of Medicine, Baskent University, Ankara, Turkey

**Keywords:** Siderosis, Iron Chelates, Hemosiderin, Deferiprone

Superficial siderosis is a rare disease and causes cerebellar ataxia, sensorineural deafness, and pyramidal tract dysfunction.^1^^,^^2^ Recurrent chronic bleeding from usually undetectable site to subarachnoid space causes hemosiderin deposition, and leads to clinical symptoms.^1^ Various disorders, such as subarachnoid hemorrhage, arteriovenous malformation, trauma, tumor, and spinal surgery precedes clinical symptoms. The interval between symptoms and the preceding disorder may take years. Magnetic resonance imaging or computerized tomography may provide clues for possible etiology like fluid-filled collection.^1^^,^^2^

I present a 63-year-old man with a complaint of difficulty walking, dizziness, and falls, with a considerable progression within one year. Neurologic examination showed motor weakness on bilateral upper limbs (4/5), proximal muscles of left leg (4/5), increased deep tendon reflex, hypoesthesia on the left side of the body, decreased vibration sense on lower limbs, bilateral extensor plantar response, wide-based stance, and ataxia while walking and sitting. He had a previous history of diabetes mellitus, hypertension, coronary heart disease, and lumbar stabilization surgery. Brain and spinal magnetic resonance imaging revealed supratentorial, infratentorial, and spinal hemosiderin deposits compatible with superficial siderosis (Figure 1). 

We could not reveal a source of active bleeding like fluid-filled collection or contrast leakage, but we thought that the vertebrate fracture was the probable site. In a few cases in literature, deferiprone, iron chelator treatment that can pass through blood brain barrier, was used as an experimental treatment.^3^ We started a dosage of 30 mg/kg/day, and scheduled follow-up for possible side effects of the drug and clinical improvement.

At the third month of the treatment, the clinical signs of the patient stabilized, and also showed improvement in motor weakness; he could sit without assistance, and walk with one cane. After 9 months of treatment, his clinical signs and symptoms were still stable.

Ninety percent of the patients with superficial siderosis, suffer from slowly progressive ataxia and hearing loss, and rarely present with seizure, episodic headache, visual loss, and olfactory impairment.^1^^,^^4^^,^^5^ The most disabling symptom is progressive gait disorder.^1^ Because of the progressive course, it is important to establish a treatment option for the patients. Surgical intervention for obliteration of active bleeding site is one of the options.

**Figure 1:**
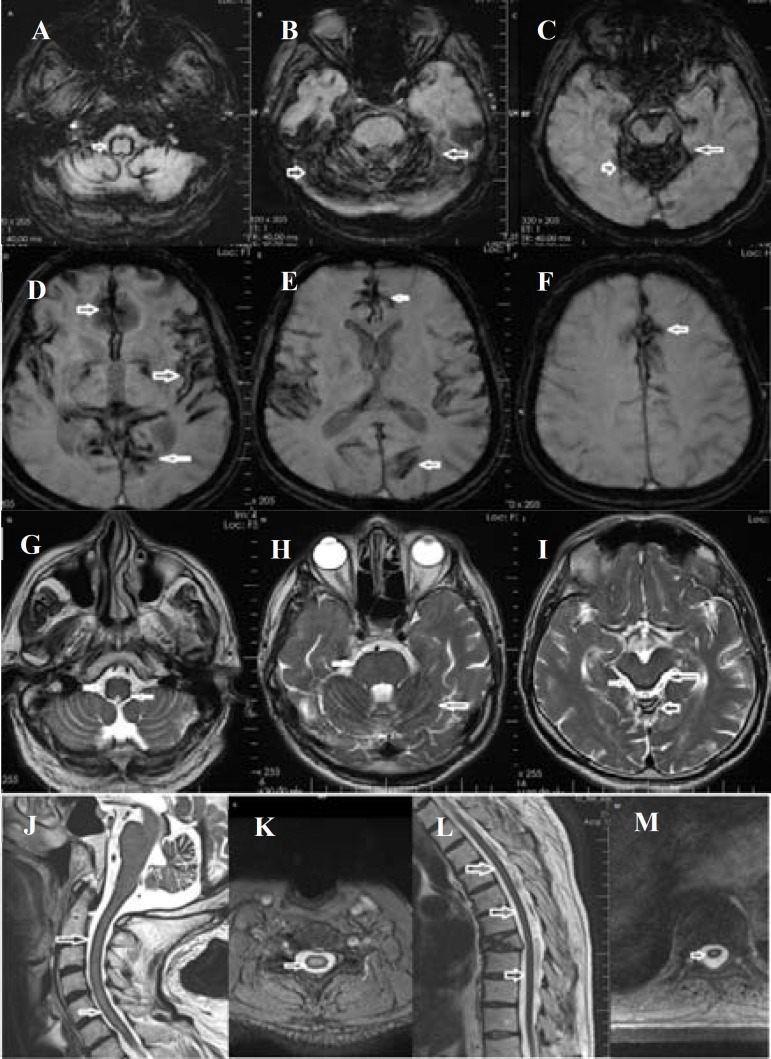
Brain magnetic resonance imaging. Susceptibility (A-F) and T2-weighted imaging (G-I) show hemosiderin deposition especially on brainstem, cerebellum (showed with white arrow). T2-weighted imaging reveals the hemosiderin deposition as hypointensity around the spinal cord as well (J-M).

Although most of the patients had red blood cells and xanthochromia in cerebrospinal fluid, active bleeding site is distinguished only in half, and surgical results are controversial.^1^^,^^5^ For patients that the site of bleeding has not been established by imaging methods, medical treatment with iron chelator deferiprone may be a promising medical choice like in our patient. There were few reports in literature about deferiprone usage in superficial siderosis as an experimental treatment.^3^ Both radiological and clinical improvement observed in treated cases, but not all. There is a risk of agranulocytosis (1-2 percent), liver enzyme elevation, and zinc deficiency.

In conclusion, we think that, in our patient, the possible underlying etiology was spinal surgery and trauma. We could not find a clue for possible bleeding site, and decided to start medical treatment. In our experience, deferiprone can be used safely with closed monitoring of possible side effects until the discovery of new and effective option for the treatment of superficial siderosis. Although the experience with the drug is based on observational case studies, randomized, placebo-controlled trials are needed to determine the exact clinic benefit.

## Notes:

**How to cite this article:** Derle E. Iron chelation in treatment of superficial siderosis. Iran J Neurol 2017; 17(4): 195-6.
